# Th1/Th2 Cytokine Ratio in Tissue Transudates from Patients with Oral Lichen Planus

**DOI:** 10.1155/2007/19854

**Published:** 2007-02-28

**Authors:** Nelson L. Rhodus, Bin Cheng, Frank Ondrey

**Affiliations:** ^1^Division of Oral Medicine and Diagnosis, Department of Diagnostic and Biological Sciences, School of Dentistry, University of Minnesota, Minneapolis, MN 55455, USA; ^2^Department of Otolaryngology, School of Medicine, University of Minnesota, Minneapolis, MN 55455, USA; ^3^Guanghua School of Stomatology, Sun Yat-Sen University, Guangzhou 510060, China

## Abstract

*Objective*. The characteristics of oral lichen planus (OLP) provoke investigators to explore possible biomarkers by which to monitor disease activity and therapeutic efficacy. Oral fluids may provide an accessible medium for analysis of such biomarkers. Previous studies have shown that activation of nuclear factor-kappa B (NF-*κ* B) plays an important role in the pathogenesis of oral lichen planus (OLP), which is a chronic inflammatory disorder mediated by T cells. Prior to the present investigation, reports of the levels of NF-*κ* B and its dependent cytokines in oral fluids have not been forthcoming. The purpose of this study was to detect the level of NF-*κ* B dependent cytokines, TNF-alpha, IL-1-alpha, IL-6, and IL-8 in tissue transudates directly from lesions of OLP, and explore the feasibility of the data for clinical application. *Study design*. Thirteen definitively diagnosed OLP subjects were enrolled in the study as were 13 age-sex matched controls. In each subject, lesion tissue transudates (TTs) were collected by a novel collection technique with a filter paper. The level of cytokines, TNF-alpha, IL-1-alpha, IL-6, and IL-8 in three types of oral fluids were determined by ELISA. *Results*. In the tissue transudate(TT), there were significantly higher level of cytokines TNF-alpha, IL-1-alpha, IL-6, and IL-8 detected in OLP patients than in controls: (TT: 40.0 ± 9.8 versus 4.5 ± 0.7, 710 ± 114 versus 305 ± 78, 150 ± 25 versus 1.7 ± 0.5, 2800 ± 260 versus 1450 ± 130, *P* < .0001; unit: pg/mL). *Conclusions*. These results indicate that NF-*κ* B dependent inflammatory cytokines may be detected at increased levels in oral lesion tissue transudates which may have diagnostic and prognostic potentials for monitoring disease activity and making therapeutic decisions in patients with OLP.

## 1. INTRODUCTION

Evidence from investigations using mouse and human models has indicated that T helper cells can be classified into at least two functional subsets, Th1 and Th2, on the basis of their cytokine profiles. Th1 cells were characterized by the production of IL-2, IFN-*γ* and TNF-*β* and were critical in the cell-mediated immunity, but Th2 cells were characterized by the production of IL-4, IL-5, IL-6, and IL-13 and played an important role in humoral immunity. These two Th subsets regulated each other's function through the antagonistic activity of their respective cytokines which determined in large extent the characteristics of immune responses and developments [1, 2]. Many studies have demonstrated a close relationship between Th1/Th2 imbalance and pathogenesis of a series of autoimmune disorders including Behcet's disease (Th1 dominant response), Sjogren syndrome secondary to hepatitis C virus infection (Th2 dominant response), psoriasis (Th1 dominant response), and others [3–5]. Increasing evidences showed that the balance of immune responses between Th1 and Th2 determined outcomes of diseases and modulation of Th1/Th2 imbalance became a new therapeutic strategy of some autoimmune diseases, for example, IL-4 in psoriasis, anti-CD4 monoclonal antibody in experimental arthritis, and IFN-*γ* in corticosteroids-resistant asthma [6–8].

Oral Lichen planus (OLP) is a chronic inflammatory disorder characterized by a T-cell-mediated immune response against epithelial cells, with persistent accumulation of T lymphocytes and epithelial cell damage. Previous studies have described the expression pattern of the different cytokines/chemokines, which involved in Th1 and/or Th2 polarization, in tissues, cultured cells, and serum from patients with OLP, including IL-2 [9–11], IFN-alpha [11–14], TNF-alpha [9, 11, 12, 15–18], IL-4 [9, 12, 18], IL-6 [11, 13, 18, 19], IL-10 [9, 12, 18], and TGF-alpha [9, 12, 20], IL-1 [18], IL-8 [21, 22]. From these results, Sugerman et al. concluded that OLP was characterized by Th1 cytokine bias [23]. This conclusion has not been supported by the study on Th1/Th2 cytokine ratio in OLP, one of the direct indicators of Th1/Th2 imbalance.

In this small pilot study, our focus was on the shift of TNF-alpha/IL-6, IL-1/IL-6, and IL-8/IL-6 ratio in tissues transduates (TT) of OLP subjects, one of the diagnostic mediums reflecting exactly local immune response of oral cavity.

## 2. MATERIALS AND METHODS

### 2.1. The clinicopathological data of subjects

All subjects were from the University of Minnesota Oral Medicine Clinic and Hospital of Stomatology, Sun Yat-Sen University. With the approval of the Committee for the Use of Human Subjects in Research, informed consent was obtained from all subjects.

Thirteen patients clinically diagnosed as OLP [24] and 13 age-sex matched healthy individuals were recruited for the study. They had to comply with the following criteria: (1) the absence of any detectable gingival and/or periodontal inflammation and other visible oral lesions, (2) the absence of history of local and systematic diseases, (3) the absence of medication record, for example, anticholinergics, antihistamines, antihypertensives, beta-adrenergic blockers, and other regimen inducing hyposalivation, and (4) the absence of previous or current treatment. Their characteristics were listed in Table 1.

The biopsy was taken from each patient, following collection of oral fluids, and the tissue specimens were processed by the Department of Oral Pathology at both universities. The pathologic diagnosis confirmed by two oral pathologists independently was consistent with clinical diagnosis.

### 2.2. Collection of tissue transudates

The lesion tissue transudates (TT) were collected by a modified technique [25]. Briefly, the TT was collected after slightly drying the OLP lesion area and then by placing a Periopaper strip directly over the area for 30 seconds. In control group, the TT was collected by the same method on the corresponding anatomic site. The volume of TT was quantified on the Periotron (Harco, Tustin, Calif, USA) and the paper strips were placed in 1.5 mL micropipette tubes containing 1 mL of saline solution. After collection, all samples were immediately stored at −20°C until assayed.

### 2.3. Detection of cytokines

Prior to analysis, a sample from the paper strips was eluted by 3 serial centrifuge extractions, each using 1 mL of PBS buffer solution at 10 000 rpm for 30 seconds, and was combined with the saline which the paper strips were initially stored in immediately after collection.

Then all samples were thawed on ice in the lab and were centrifuged at 6 000 rpm for 20 minutes. Supernatants were drawn off and used in the ELISA cytokine assays. For Each sample, 100 *μ*L supernatant was used for these assays.

TNF-alpha, IL-1-alpha IL-6, and IL-8 concentrations were determined by the quantitative sandwich ELISA technique as previously described [26], using commercially available kits (R&D Systems Inc., Minneapolis, Minn, USA) with a sensitivity of less than 3 pg/mL. Briefly, Costar 96-well EIA Plates were coated with a 1 : 1000 dilution of 1 mg/mL anti-IgA (Dako). Plates were incubated overnight. Standards, samples, and blanks were incubated for 2 hours. Sample dilutions ranged from 1 : 50000 to 1 : 200000 and standards ranged from 0.5 to 10 ng/mL (Sigma). Biotinylated antibodies for each cytokine were incubated for 2 hours. Avidin-alkaline phosphatase conjugate (Organon Teknika, The Netherlands) was incubated for 2 hours. Phosphatase substrate was added and was allowed to develop for 30 minutes in the dark and reaction was stopped with 1 mol/L NaOH. All incubations were at 37°C. All plates were washed between each step with PBS-Tween wash buffer. Absorbance was measured at 450 nm using software from the Molecular Devices Corporation. The standard curves were plotted on a log-log scale. Sample concentrations were determined by linear regression of log average column absorbance against log standard concentration. R-squared values for typical log-log standard curves were >0.98; column coefficients of variation ranged between 10%–20%. Correlations between sample dilutions were >0.90.

### 2.4. Statistics

Data were expressed as mean ± standard deviation (SD). Since it was not clear whether the data were distributed normally, the test of data normality (Shapiro-Wilk test), Mann-Whitney test, and independent-samples *t* test were performed, respectively. *P* < .05 was considered to be statistically significant. Statistical Package for Social Studies (SPSS, Version 11.0, SPSS Inc., Chicago, Ill, USA) software was used to analyze the data.

## 3. RESULTS

All data were confirmed to be normal distribution by Shapiro-Wilk test. The same results were obtained from both nonparametric and parametric tests. The statistical results of independent-samples *t* test were presented in Tables 2 and 3.

## 4. DISCUSSION

The Th1/Th2 balance hypothesis emerged in the late 1980s. Currently, much of the literatures have elevated the Th1/Th2 balance concept to the level of paradigm, moreover cytokine and anticytokine therapies, derived from the concept of modulating Th1/Th2 imbalance, have been successfully applied to several autoimmune diseases [27], including Sjogren syndrome [28, 29], psoriasis [6], and corticosteroids-resistant asthma [8]. Yet some observations indicated that addressing the role of individual cytokines during immune response may be far more informative than using the blanket Th1/Th2 descriptors [2]. In OLP, an inflammatory oral mucosal disorder mediated by T cells, however, several lines of studies on individual cytokines introduced above seem not able to outline the general immune response in OLP and not to give some new insights into the therapy strategy of OLP so that the application of cytokine and anticytokine therapies to OLP seems to have long way to go. Thus, it is necessary to investigate the possible Th1/Th2 pattern in OLP which will be beneficial to its treatment.

Traditionally, IL-2 and IFN-*γ*, IL-4 and IL-5 were classified into Th1 cytokine and Th2 cytokines, respectively. As more and more cytokines have been described and characterized, some cytokines that were not necessarily secreted by CD4^+^ T cells promoted the development of either Th1 or Th2 cells [2]. For example, TNF-alpha, IL-1, and IL-8 as Th1-associated cytokines and IL-6 as Th2-associated cytokines have been assigned in some investigations [30, 31]. In this preliminary study, TNF-alpha, IL-1, IL-8, and IL-6 were assigned to Th1 and Th2 cytokines, respectively. The level of TNF-alpha, IL-1-alpha, IL-6 and IL-8 in TT of OLP patients and normal controls was determined. Our results showed that the concentration of TNF-alpha, IL-1-alpha, IL-6, and IL-8 was significantly higher in TT of OLP patients than in that of controls, furthermore all ratios of cytokines, TNF-alpha/IL-6, IL-1/IL-6, and IL-8/IL-6 in OLP patients were decreased significantly, compared with that of controls. This result is, to our knowledge, the first to suggest the presence of a Th2 cytokine-dominant condition in these limited subjects of OLP. Since previous studies indicated Th1 cytokine bias in OLP [23], it seems to be necessary to evaluate carefully the published data before a conclusion will be made.

In the past analysis on simultaneous expression of Th1 and Th2 cytokines, Simark-Mattsson et al. noted that cells expressing mRNA for IL-2, IL-4, IL-10, TNF-*α*, and TGF-beta 1 were found in all the biopsies studied, and message for IFN-*γ* and IL-10 was detected in the cultured T-cell lines of all the patients [9]. Fayyazi et al. revealed the coexpression of IFN-*γ* and IL-6 mRNA in the upper subepithelial connective tissue and basal and suprabasal keratinocytes of altered skin/oral mucosa [13]. Khan et al. reported that unstimulated OLP lesional T cells secreted IFN-*γ* in vitro but IL-4, IL-10, and TGF-beta 1 secretion were not detected [12]. Yamamoto et al. detected increase of IL-4 and TNF-alpha in serum from about one third of the patients, and IL-6 in all patients [11, 32], and they also found that more IFN-alpha, IL-6, and TNF-alpha were generated by tissue-infiltrating mononuclear cells (TIMCs) of OLP, compared to TIMC from the chronically inflamed gingivae [17]. But these studies had not calculated and compared the Th1/Th2 cytokine ratio, the direct evidence of Th1/Th2 balance, in OLP.

Similarly, our detection also showed the simultaneous argumentation of TNF-alpha, IL-1-alpha, IL-8, and IL-6 in TT of OLP patients. However, the decreased ratio of cytokines, TNF-alpha/IL-6, IL-1/IL-6, and IL-8/IL-6, was measured in OLP patients. It may be attributed to the reason that the increase of IL-6 was far more extent than that of TNF-alpha, IL-1-alpha, IL-8 as seen in Table 2. This result indicated the possibility that a Th2-dominated immune response does occur in a subgroup of OLP patients. Moreover, TT was selected as an analytic medium in our study. With the advantages of safety, minimal invasion, and easy access, several types of body fluids have been applied to a lot of cytokines analyses in clinical and experimental studies, including bronchoalveolar lavage [33, 34], urine [35], synovial fluid [36, 37], and saliva [38, 39]. Since a series of cytokines produced in tissues of OLP through autocrine and paracrine processes plays important roles in the local inflammatory response of OLP [23], the collection and analysis of TT will avoid the interference of subclinical inflammatory lesions of oral-facial region. The results from TT analysis may more exactly reflect the local immune situation of OLP lesion than that from whole saliva, gingival crevicular fluid, and other oral fluids. Therefore, our findings presented here did give the evidence that a Th2 cytokine-dominanted immune response exists in some cases with OLP.

In summary, our study, together with the previous reports, indicated that the different patterns of Th1/Th2 imbalance, Th1 or Th2 overactivation or mixed Th1/Th2 conditions, may occur in the pathogenesis of OLP. The large scale of patients and more types of cytokines should be included in the future researches before the available results will be used in monitoring disease activity and direction of OLP therapy.

## Figures and Tables

**Table 1:** Clinical features of subjects.

	OLP (*n* = 13)	Control (*n* = 13)

Age (y)
Mean ± SD	57 ± 11	58 ± 12
Range	42 ∼ 68	40 ∼ 74
Gender
F/M	13/0	13/0
Clinical classification
Erosive	9	—
Reticular and/or plaque-like	2	—
Atrophic	2	—
Site
Buccal mucosa	11	—
Buccal mucosa and gingiva	2	—
Histological classification
OLP	12	—
OLP and mild dysplasia	1	—

**Table 2:** The Levels of four cytokines in TT from subjests (pg/mL).

Group	TNF-alpha	IL-1-alpha	IL-6	IL-8

OLP (*n* = 13)	40.0 ± 9.8	710 ± 114	150 ± 25	2800 ± 260
C (*n* = 13)	4.5 ± 0.7	305 ± 78	1.7 ± 0.5	1450 ± 130
*t* value	13.23	10.60	21.44	16.80
*P* value	< .0001	< .0001	< .0001	< .0001

**Table 3:** Th1/Th2 cytokine ratio in TT.

Group	TNF-alpha /IL-6	IL-1/IL-6	IL-8/IL-6

OLP (*n* = 13)	0.3 ± 0.1	4.8 ± 0.9	19 ± 3
C (*n* = 13)	3.0 ± 1.0	200 ± 78	947 ± 273
*t* value	9.671	9.034	12.255
*P* value	.0001	.0001	.0001
